# Bridging the Digital Divide Among Racial and Ethnic Minority Men Who Have Sex With Men to Reduce Substance Use and HIV Risk: Mixed Methods Feasibility Study

**DOI:** 10.2196/15282

**Published:** 2020-04-29

**Authors:** Elaine Hsiang, Claudine Offer, Maximo Prescott, Amy Rodriguez, Emily Behar, Tim Matheson, Diane Santa Maria, Glenn-Milo Santos

**Affiliations:** 1 School of Medicine University of California San Francisco San Francisco, CA United States; 2 Substance Use Research Unit Center for Public Health Research San Francisco Department of Public Health San Francisco, CA United States; 3 Cizik School of Nursing University of Texas Health Science Center at Houston Houston, TX United States; 4 Department of Community Health Systems School of Nursing University of California San Francisco San Francisco, CA United States

**Keywords:** ecological momentary assessment, men who have sex with men, text messaging, substance use, HIV, digital divide, focus group

## Abstract

**Background:**

Ecological momentary assessment (EMA) is a promising data collection tool for mobile health interventions targeting episodic health behaviors. For substance-using men who have sex with men (SUMSM), EMA is becoming more widely utilized in efforts to characterize substance use and sexual risk factors for HIV transmission. However, recent literature demonstrates emerging concerns over compliance and lower EMA engagement and data concordance among racial and ethnic minority SUMSM.

**Objective:**

This study aimed to provide a qualitative evaluation of the barriers and facilitators of EMA as a data collection tool among racial and ethnic minority SUMSM.

**Methods:**

Between October and November 2017, 45 racial and ethnic minority SUMSM were recruited from a list of prior research participants at the San Francisco Department of Public Health to participate in daily EMA surveys on their substance use and sexual health behaviors for 1 week, followed by in-person focus groups (FGs). A total of 4 FGs explored the participants’ experiences with the surveys, issues regarding privacy and confidentiality, and suggestions for improvement. Qualitative analysis was performed using content analysis. Descriptive statistics and Fisher exact tests were used to assess the associations between demographics or substance use behaviors and EMA completion.

**Results:**

Overall, 93.9% (295/314) of all delivered surveys were initiated, and of those, 98.0% (289/295) were completed. Neither participant demographics, including race (*P*=.65) or age (*P*=.43), nor substance use behaviors, including the frequency of alcohol (*P*=.40) or methamphetamine (*P*=.91) use or any cocaine (*P*=.28), crack (*P*=.99), or polysubstance use (*P*=.24), were found to be associated with survey completion. Overall, participants were receptive to the text message–based EMA surveys. Facilitators included survey timing, user-friendly survey design, survey-stimulated self-reflection, coding of sensitive phrases, and other privacy benefits of a mobile survey. Barriers included an inability to correct texting errors and participants’ perception of judgment or stigmatization related to questions about condomless sex. To improve EMA compliance and uptake, participants suggested adding response confirmations, clarifying survey language, and continuing to diversify the study audience.

**Conclusions:**

EMA appears to be feasible and acceptable among this sample of racial and ethnic minority SUMSM. Close attention to EMA study design and the development of nonjudgmental, contextualized questions regarding stigmatized health behaviors may be critical to further improve EMA compliance.

## Introduction

In the United States, men who have sex with men (MSM) experience higher rates of substance use compared with the general population [1-3]. A growing body of literature independently links binge drinking, methamphetamine, and injection drug use to sexual risk behaviors and HIV transmission among MSM [2,4]. Although the incidence of HIV in MSM with injection drug use decreased slightly between 2012 and 2016, MSM remained the single largest demographic, accounting for 70% of all new HIV infections in the country (70%) [2]. HIV continues to disproportionately impact the health of substance-using men who have sex with men (SUMSM) [5-7] as well as MSM who identify as racial and/or ethnic minorities [8-11]. It is unclear whether substance use is associated with racial disparities among SUMSM living with HIV [12,13].

Understanding the prevalence, patterns, and frequency of substance use in SUMSM is necessary for the development of effective interventions to address substance use and HIV infection in this population. This research often relies on self-report of substance use behaviors and is vulnerable to social desirability bias, limitations in recall ability, and other mechanisms that introduce variations in validity [14,15]. In addition, lower data reliability among racial and ethnic minorities has been associated with the fear of legal repercussions because of the disproportionate criminalization of substance use, particularly among black/African American adults [12,16-19].

Ecological momentary assessment (EMA) has emerged as a promising tool for substance use and mobile health intervention research. EMA utilizes mobile technologies such as text messaging to collect real-time data and can reduce recall bias when characterizing substance use and other episodic health behaviors [20]. The tremendous potential of EMA as a robust data collection method is associated with the widespread ownership and use of mobile devices [21,22], and EMA has been leveraged in a number of studies describing substance use patterns, sex events, and the delivery of health interventions [23-27]. Despite its many benefits, EMA remains underutilized in substance use research focused on sexual and gender minorities, including SUMSM. In addition, concerns about response compliance feature prominently in the EMA literature, with a recent meta-analysis of EMA studies related to substance use reporting a pooled compliance rate lower than the recommended 80% [28]. Beyond evidence that compliance may differ between those with and without a clinical diagnosis of a substance use disorder [28], few studies have explored EMA engagement among SUMSM [29-31] and potential sociodemographic correlates. Previous studies by our research group found a significantly lower adjusted odds of responding to EMA text messages among racial and ethnic minority participants [32] as well as lower concordance in methamphetamine and alcohol reporting via EMA compared with data provided on timeline follow-back assessments [33]. These findings provide limited data to suggest a digital divide between racial and ethnic minority SUMSM and white SUMSM with regard to data reporting in EMA.

As EMA becomes more widely employed in substance use literature among SUMSM, it is important to explore the differences in EMA engagement and data concordance between white and racial and ethnic minority SUMSM and develop strategies to ensure that racial and ethnic minority SUMSM can fully benefit from interventions utilizing EMA data to reduce substance use and related HIV risk factors. Involving racial and ethnic minorities in feasibility and acceptability studies is a critical step in this process, yet few studies have done so [29,30]. Therefore, this study aimed to evaluate the acceptability of a text message–based survey leveraging EMA data among a sample of racial and ethnic minority SUMSM and elucidate barriers to and facilitators of EMA engagement and utilization.

## Methods

### Participant Recruitment and Data Collection

The Digital Divide study recruited 45 participants over phone in October 2017 using a list of previous SUMSM study participants at the San Francisco Department of Public Health who were willing to be contacted for additional studies. Eligibility was limited to participants living in the Bay Area who identified as men, were aged 18 years or older, belonged to a racial and ethnic minority, reported having sex with men, and were using at least one of four target substances (alcohol, methamphetamine, cocaine, and/or crack). Participants were required to be English speaking, have a phone that could receive and send text messages, and agree to participate in a focus group (FG). All participants provided informed consent. Study procedures were reviewed and approved by the Institutional Review Board at the University of California, San Francisco (CHR 17-22897).

**Figure 1:**
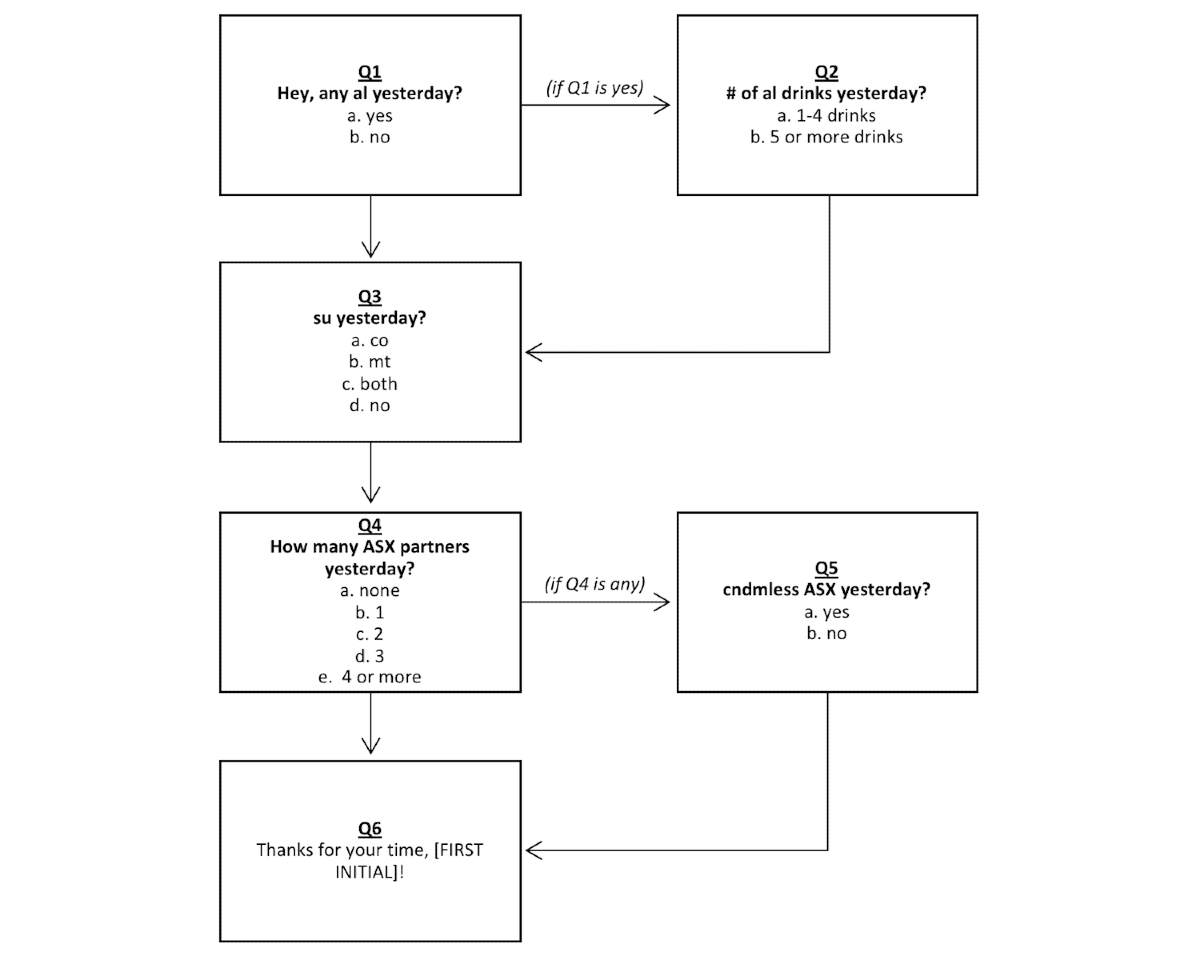
Daily text message ecological momentary assessments for substance-using men who have sex with men. Abbreviations: al: alcohol; ASX: anal sex; cndmless: condomless; co: cocaine; mt: methamphetamine; Q: question; su: substance use.

At baseline, a phone survey was conducted to collect the participants’ demographic and substance use information. Participants were asked to quantify their frequency of alcohol use, binge drinking, cocaine use, crack use, and methamphetamine use in the previous 6 months. A 6-month recall period is a validated recall window used in prior epidemiological studies to gather self-reported substance use data in a feasible manner [34,35]. Once enrolled, participants received a confirmatory email with the date and time of their FG, an instructional guide to the text message surveys, and individualized technical support. Participants then received text message surveys on 7 consecutive days. Surveys were routed through the Health Insurance Portability and Accountability Act-compliant CareSpeak mobile health platform (OptimizeRx Corp). The surveys included 3 to 5 questions (depending on each individual’s self-reported behaviors on a given day) and were estimated to take less than 5 min (Figure 1). Abbreviations (eg, *al* for alcohol, *su* for substance use, and *asx* for anal sex) were used in text message prompts to provide confidentiality, and a key was provided in the initial guide. Participants had a choice between receiving the text messages at the default time of 10:30 AM each day or another time of their choice. All except one participant chose to receive messages at the default time.

After the weeklong EMA study, all 45 participants attended 1 of the 4 FGs in November 2017. There were 8 to 15 participants per FG, each lasting between 1.5 to 2 hours. A semistructured interview guide was designed to explore the participants’ experiences with the surveys (Multimedia Appendix 1). Predefined topics included general barriers and facilitators to initiating or completing the daily text message surveys; suggestions for improving survey delivery, questionnaire design, and incentives; and issues with privacy or confidentiality. FGs were conducted by trained research staff who identified as MSM and formerly used methamphetamine.

Participants received US $2 for each text message survey that was completed and a bonus of US $6 for completing all 7 surveys. Participants were paid US $70 for attending the FG. Hence, participants received up to US $90 for completing all the study procedures.

### Analytic Approach

#### Qualitative Data

The FGs were audiorecorded and transcribed verbatim. The transcripts were coded using Dedoose 8.0.35 (SocioCultural Research Consultants, LLC). Data saturation, in which additional data points do not contribute new information, was attained in the 4 FGs [36]. Content analysis was used to develop a codebook of emerging themes and perform subsequent analyses [37]. Two of the authors coded the transcripts and conferred frequently to discuss emerging themes and develop additional codes or resolve disagreements. Coded excerpts were then extracted, organized by category, and reviewed iteratively and corroborated by a third author before thematic compilation.

#### Quantitative Data: Demographics

Frequency of alcohol use, as well as binge drinking, was assessed using 4-point scales; however, the use of cocaine, crack, and methamphetamine was recorded as any or no use in the past 6 months because of the small sample sizes across the frequency categories. A variable for polysubstance use was generated to categorize the number of aforementioned substances the participants had used in the past 6 months (*only 1 substance* or *2 or more substances*).

#### Quantitative Data: Survey Completion

The primary outcome of interest was the completion of all text messaging surveys, dichotomized as successfully or unsuccessfully completing all 7 daily surveys. The completion of a survey required participants’ responses to all applicable questions; surveys in which participants responded to at least one question but did not reach the final text thanking them for their time were classified as initiated but not completed. Owing to a technical issue, 1 participant received only 6 surveys in total; the authors considered this participant’s completion of the 6 surveys as successful completion of all surveys. Descriptive analyses and Fisher exact tests were conducted to evaluate the associations between demographics or substance use behaviors and the completion of EMAs. Analyses were performed with Stata 14 (StataCorp), and statistical significance was determined using a *P* value of <.05. Given the scope of the study and the intent to elucidate baseline correlates of survey completion as a measure of feasibility and acceptability, the substance use and sexual behavior data obtained from the EMA surveys themselves were not analyzed as outcomes.

## Results

### Participant Characteristics

Demographics and substance use behaviors collected from the baseline phone surveys are presented in Table 1. Participants (n=45) had a mean age of 44 years (median 43 years; range 21-71 years). All participants reported being cisgender men (ie, people assigned male sex at birth and currently identifying as men). The majority of participants were African American (24/45; 53%) or Hispanic/Latino (16/45; 36%) and had sex exclusively with men in the past 6 months (35/45; 78%). Most (35/45; 78%) SUMSM in our sample reported using at least two substances in the past 6 months. The most commonly used substance was alcohol (40/45; 89%), followed by methamphetamine (31/45; 69%). The frequency of methamphetamine use was relatively high, with 22% (10/45) of all participants reporting methamphetamine use on 2 or 3 days per week and 27% (12/45) reporting use on 4 or more days per week in the past 6 months. Recent use of cocaine was more common (12/45; 27%) than crack (5/45; 11%).

**Table 1:** Participant demographics, sexual behaviors, and substance use behaviors collected at baseline (n=45).

Characteristics		Participants, n (%)
**Age (years)**		
	18-24	1 (2)
	25-34	9 (20)
	35-44	14 (31)
	45+	21 (47)
**Race/ethnicity**		
	Asian/Pacific Islander	3 (7)
	Black/African American	24 (53)
	Hispanic/Latino	16 (36)
	Mixed	2 (4)
**Number of sexual partners in the past 6 months**		
	1	3 (7)
	2-5	15 (33)
	6 or more	27 (60)
**Gender of sexual partners in the past 6 months^a^**		
	Only men	35 (78)
	Multiple	10 (22)
**Frequency of alcohol use in the past 6 months**		
	None	5 (11)
	Once a week or less	8 (18)
	2-3 days per week	23 (51)
	4 or more days per week	9 (20)
**Frequency of binge drinking in the past 6 months**		
	Never	13 (29)
	Once a month or less	12 (27)
	Weekly	17 (38)
	Daily or almost daily	3 (7)
**Cocaine use in the past 6 months**		
	Not used	33 (73)
	Used at least once	12 (27)
**Crack use in the past 6 months**		
	Not used	40 (89)
	Used at least once	5 (11)
**Methamphetamine use in the past 6 months**		
	Not used	14 (31)
	Used at least once	31 (69)
**Frequency of methamphetamine use in the past 6 months**		
	None	14 (31)
	Once a week or less	9 (20)
	2-3 days per week	10 (22)
	4 or more days per week	12 (27)
**Number of substances used in the past 6 months**		
	1	10 (22)
	2 or more	35 (78)

^a^Participants were not asked whether their sexual partners were cisgender or transgender.

### Survey Compliance

Over the 7-day sampling frame, 314 text message surveys were successfully delivered to the participants. Of these 314 surveys, 295 (93.9%) were initiated. Overall, 9 of the 19 (47%) noninitiated surveys were attributable to 2 participants alone; one of the participants temporarily misplaced his or her phone for part of the study and another did not initiate any of the surveys. Among the 295 surveys that were initiated, 289 (98.0%) were completed. The baseline data of the participants completing all 7 text messaging surveys in their entirety are presented in Table 2. Neither the participants’ race/ethnicity (*P*=.65) and age (*P*=.43) nor any of the substance use behaviors, including frequency of alcohol (*P*=.40) or methamphetamine use (*P*=.91) or any cocaine (*P*=.28), crack (*P*=.99), or polysubstance use (*P*=.24), were significantly associated with the completion rates.

**Table 2:** Demographics of participants (n=45) based on the completion of all 7 text messaging surveys.

Demographics		Completed^a^ (n=32), n (%)	Did not complete (n=13), n (%)	*P* value
**Race**				**.65**
	Asian/Pacific Islander	3 (100)	0 (0)	
	Black/African American	17 (71)	7 (29)	
	Hispanic/Latino	10 (63)	6 (37)	
	Mixed	2 (100)	0 (0)	
**Age (years)**				**.43**
	18-24	1 (100)	0 (0)	
	25-34	5 (56)	4 (44)	
	35-44	9 (64)	5 (36)	
	45+	17 (81)	4 (19)	
**Number of sexual partners in the past 6 months**				**.99**
	1	2 (67)	1 (33)	
	2-5	11 (73)	4 (27)	
	6+	19 (70)	8 (30)	
**Gender of sexual partners in the past 6 months^b^**				**.99**
	Only men	25 (71)	10 (29)	
	Multiple	7 (70)	3 (30)	
**Frequency of alcohol use in the past 6 months**				**.40**
	None	5 (100)	0 (0)	
	Once a week or less	6 (75)	2 (25)	
	2-3 days per week	14 (61)	9 (39)	
	4 or more days per week	7 (78)	2 (22)	
**Frequency of binge drinking in the past 6 months**				**.64**
	Never	11 (85)	2 (15)	
	At least once a month	8 (67)	4 (33)	
	Weekly	11 (65)	6 (35)	
	Daily or almost daily	2 (67)	1 (33)	
**Use of cocaine in the past 6 months**				**.28**
	Not used	25 (76)	8 (24)	
	Used at least once	7 (58)	5 (42)	
**Use of crack in the past 6 months**				**.99**
	Not used	28 (70)	12 (30)	
	Used at least once	4 (80)	1 (20)	
**Use of methamphetamine in the past 6 months**				**.99**
	Not used	10 (71)	4 (29)	
	Used at least once	22 (71)	9 (29)	
**Frequency of methamphetamine use in the past 6 months**				**.91**
	None	10 (71)	4 (29)	
	One a week or less	6 (67)	3 (33)	
	2-3 days per week	8 (80)	2 (20)	
	4 or more days per week	8 (67)	4 (33)	
**Number of substances used in the past 6 months**				**.24**
	Only 1 substance	9 (90)	1 (10)	
	2 or more substances	23 (66)	12 (34)	

^a^Initiated but unfinished surveys were not considered *completed*.

^b^Participants were not asked whether their sexual partners were cisgender or transgender.

### Qualitative Results

Overall, participants expressed ease with and receptivity of the text message–based EMA surveys. The 3 overarching themes of barriers to survey completion, facilitators of survey completion, and suggestions for study improvement covered a variety of aspects related to participants’ experiences with the surveys. Selected quotes are illustrative of both the range of experiences as well as common perspectives.

#### Facilitators

##### Survey Scheduling and Delivery

FG discussions highlighted the consistent, timely scheduling of survey delivery as a major strength of text messaging as a survey modality. Over the course of the study, some participants reported overcoming an initial hesitance about the reliability of texted surveys:

I was like, maybe it’s going to come through at 10:30 today, and then tomorrow it’s going to come in at like 10:45 or something like that. And because there’s always glitches in technology, but—for the seven days, when—every day when it came through at the same time, I was like, you know what? Someone nailed it on the head.

FG1—Participant M

The ability to customize the time of the survey delivery provided a sense of ownership and empowerment, but many agreed that 10:30 AM granted a sufficient timeframe to respond to the surveys. The participants also appreciated the ability to incorporate the surveys into their daily routine and ensure completion of as many surveys as possible:

I like the option of being in power...being able to choose the time I wanted it to actually come, that makes me feel like I’m more in control of it.

FG4—Participant M

Some participants explained that having a full day to respond to the surveys allowed them to wait and do so when they felt they could give accurate answers. For example, if participants received survey prompts when they were using substances, they could decide whether they had enough capacity to complete the surveys then or later:

I was usually “unavailable” (laughing). But that’s the truth. I was tripping. During the time I was kind of high. But I was coherent [on the survey], because this was business.

FG3—Participant F

##### Survey Design

The flexibility of not having to complete the survey in one sitting was frequently characterized as a user-friendly feature. Participants commented on the value of being able to return to the surveys at a more convenient time, especially for those unable to respond during work hours. Many found this to be a benefit even when the surveys took little time to complete:

Because you have to be in that professional environment, and I’m getting these text messages while I’m trying to do work...a couple of times I did one or two questions and then at the very end, like at 5 o’clock in the afternoon, I was like, “oh shit, oh shit, better go and check that thing again,” and there it was still.

FG3—Participant D

One participant described how participating in the surveys changed his relationship with his mobile phone. The ease of completing surveys with just a few keystrokes, combined with the responsiveness of the automated system, helped this participant become more comfortable with texting:

Here comes another question, and then you answer it; about 30 seconds later, it beeps, “Thank you for your time!” You know, it was great. I actually like texting more—I hated it before—and now I actually don’t hate it. So this has actually converted a person who hates texting to someone who is open to it.

FG1—Participant F

Participants agreed that the survey was of an appropriate, and even optimal, length. Many participants could complete the survey within 5 min. One FG discussed the optimal number of questions. Although most said that they preferred the current number of 3 to 5 questions, others expressed a willingness to complete up to 10 questions at a time. Across FGs, it became evident that the speedy delivery of questions with defined, unambiguous answer choices was a stronger contributor to a positive experience than the actual number of survey questions:

It was very quick, and it seemed as though, like, the categories [had] a nice gap within it...So it gave you room if you was moderate, or social, or what have you.

FG2—Participant P

##### Personalization and Privacy

The personalization of texts using participants’ initials (Figure 1) was often cited as a positive aspect of the surveys. Participants felt that they were being addressed personally and engaged in conversation, rather than simply filling out a form. Many appreciated the conversational language, stating that an interactive experience complemented the text messaging format and made them more likely to respond, as if they were texting with a live recipient:

At the very end, when they said, “Thank you for your time,” I said, “No problem” (laughing).

FG1—Participant M

Several participants mentioned that they preferred being addressed by their initials, rather than their full name, because of increased privacy. Privacy was important to most participants when answering questions about sexual activity and substance use, but opinions differed with regard to other privacy safeguards in the survey, particularly the abbreviations used in the text messages. Although some felt that the abbreviations should have been spelled out to improve readability, participants ultimately agreed that having a code for terms such as *methamphetamine* or *condomless anal sex* protected their privacy:

[The abbreviations] were just for you, because you knew what they were...but somebody just picking up your phone and looking, they’d never know.

FG3—Participants F and U

I do [need codes] if you’re living with a significant other. This could break up our relationship, marriage, such-and-such.

FG1—Participant F

Another participant shared his preference for coded messages because of past experiences with security breaches:

I need that option, too, because sometimes my phone has been tapped in the past.

FG2—Participant G

Participants spoke of completing surveys on their personal phones as a major facilitator of both privacy and convenience. Compared with in-person questionnaires, surveys that could be completed alone or in any chosen location minimized the stress and stigma of giving truthful answers:

I appreciated the ease...it wasn’t really about anybody looking over my shoulder. So, I just appreciated that it was short, it was easy, and I could do it on the run, or waiting for a bus, or doing it at work.

FG4—Participant E

##### Facilitator of Personal Reflection

A number of participants reported that participating in the study provided a beneficial exercise in understanding their own substance use behaviors. Some individuals were surprised after quantifying their substance use on a day-to-day basis:

It also helped me self-reflect, because it was a busy week. And I was like, damn, I’ve been drinking a lot...Because I kept answering each day how many drinks I’ve had then, so it kind of kept me in check.

FG3—Participant C

For 1 participant, the reflective benefits of the surveys extended beyond individual substance use patterns. Recognizing that text messaging was a relatively new modality in collecting substance use data, this participant felt that he could help make a meaningful contribution to public health research:

It taught me [to] be more complete with things. It actually teaches you a few things, this survey. It opens up your mind. It’s not like just, “Okay, let’s go get this money.” There’s more to it. I made it more useful and utilized it in credence to some kind of meaning in my life...[helping] set a precedence for the rest of America, for the rest of any other public health service.

FG1—Participant A

Other participants commented on the deliberate representation of racial and ethnic minority MSM in this study and the impact of seeing other racial and ethnic minorities participate in research:

I like the galvanizing of people, gathering the vibes, and gathering the tribes of San Francisco. I like the connectivity...You can come to a focus group, and you’re like, “Okay, where is everybody at that’s supposed to be there?” It’s good to see an actual turnout for some focus group to actually see more than one or two faces other than the people who are supposed to be there. It brings back some hope. Like, you’re giving me back some hope, thinking that maybe there is a difference that you’d be making.

FG1—Participant E

#### Barriers

##### Inability to Correct Errors

The major barrier to accurate and complete documentation of participants’ responses was the inability to change the previously submitted responses. Although this was not identified as a barrier to engagement, participants spoke widely of the impact of typographical errors (typos) on their survey responses. Participants discussed common experiences where either mistyping a letter or the autocorrect feature on their mobile phones resulted in a different answer choice than intended:

I would like constantly just do typos, and I was just wondering like, instead of “B,” I put a “C.” Was there any way to fix it?

FG1—Participant J

Another individual described a situation where he had incorrectly recalled his substance use from the day prior:

There were a couple of times that I put the wrong answer...And when I first wake up, and I get that survey, I remember answering the questions, but not remembering, “oh, I did have a glass of wine last night. Why did I say no?” And I couldn’t change the answer once I submitted it.

FG3—Participant B

Participants also highlighted some confusion over response options. For survey questions structured to receive *yes* or *no* responses, some submitted *Y* or *N* instead of the provided response options *A* or *B*, respectively, and found themselves unable to verify their answers or correct the errors:

I kept putting “Y” or “N” for answering the questions because it was just what I was used to. The whole “A,” “B,” “C” part—sometimes I kind of got confused for a minute.

FG3—Participant C

In addition, unrecognized survey responses occasionally prevented the display of subsequent questions, thus truncating the surveys unless a CareSpeak representative manually texted the participants to input a valid response.

##### Sensitive Topics

The FGs drew mixed responses when asked about questions they would have preferred to skip. Not all participants felt comfortable answering all the survey questions. One participant described feeling judged for his responses on condomless anal sex:

It’s an insecurity thing, you know. I’m condomless most of the time, so, it just kind of brings out self-doubt. It’s one of those things—because I’m aware and it’s kind of looked down upon.

FG2—Participant A

Multiple participants from this FG responded that because they regularly engaged in condomless sex or assumed sex would be condomless, the goals of this question were unclear and risked bringing up unpleasant or unwanted memories:

It’s funny, because that’s a stupid question to ask me. That’s what I would see from it, honestly. Like, why you asking that?

FG2—Participant R

The questions forced you to remember things that you—probably wanted to forget.

FG4—Participant B

Other participants felt that the substance use questions were subtly structured to screen for substance use disorders, and were reluctant to respond without the ability to provide context:

I actually thought that the survey was trying to see how much you really drink. You know, to see, “oh well, she’s an alcoholic,” or “he’s an alcoholic.” That’s what I thought that’s what the survey was doing.

FG4—Participant A

In response to this discussion, 1 individual expressed his frustration that others would skip or fabricate answers:

I understand what the question is, but once again, you’re an adult and you signed up for this, and you knew what you were signing up for...So there’s no way you should be saying, I don’t want to answer this question or that question because you explained it from the beginning, what the survey is all about. So how can you backtrack now? But we’ll backtrack some of that money that you get and everything will be okay. Put it to them that way.

FG3—Participant U

#### Suggestions

##### Additional Survey Features to Improve Usability

Given the participants’ earlier discussions on errors in response, many felt that the survey could be improved by adding a final review and confirmation of answers before submission:

Some people are really trying to be as honest as possible when they’re trying to respond to these messages, and if they respond with the wrong answer and they want to go back and change it, I think that makes sense to summarize it at the end and see if all the answers are correct.

FG2—Participant S

Another suggestion included sending a *bump* to remind participants to complete the survey:

I don’t want to disrespect your study, but your text was deprioritized to me. Yeah, whether you pay me or not, I was like, “Yeah, I want to do this because I committed to it.” And when I commit to something, it’s like a job and I want to, you know, do my best to complete the assignment. But, honestly, I have higher priorities...I get an average of about sixty emails and, probably twenty texts on average in a day...That’s why having a reminder would be helpful.

FG4—Participant J

Participants commented on the large volume of texts, emails, and push notifications they received each day, debating whether a reminder text, email, or even call would increase the survey completion rates or add to their notification burden and contribute to survey noncompletion.

##### Clarifying Abbreviations

Participants brainstormed ways to facilitate the uptake of the abbreviations used in the study, especially after their peers raised concerns regarding privacy. As the abbreviation key was emailed to participants days to weeks before the start of the study, many reported having to search through their emails when attempting to complete the first survey:

The first text was just a question right away. So, there was no kind of priming. Even though I was primed a week ago, I totally forgot about it.

FG4—Participant N

Therefore, one suggestion was to include the survey instructions and abbreviation guide within the text message surveys themselves:

That way I can see, “Oh, okay, this is what I’m about to do. These are the codes.” And then I can begin to answer the questions. But that would be super, super easy. I don’t see how anyone could be confused that way, if it’s embedded in the first question.

FG3—Participant C

Other suggestions included streamlining the abbreviation format by uniformly using uppercase or lowercase (eg, rather than the *asx* used for anal sex and *su* for substance use) to prevent confusion between abbreviations and acronyms as well as using alternative codes altogether. One FG discussed using emojis to represent different substances or sex behaviors, such as an unpeeled banana for condomless sex, sugar or salt for cocaine, and a cloud or blowing wind for methamphetamine.

##### Broadening the Study Audience and Focus Group Outreach

The FGs highlighted the need to continue recruiting diverse participants to increase the uptake of text messaging survey among MSM. Diversity extending beyond race and even sexual orientation or behaviors featured prominently in the dialog:

One thing I would love: it’s good to come together, and get a gathering of the minds or consensus of what we all were just involved in, and then it also shows that it’s not just one age group or a demographic that is participating; it’s showing that it’s not just us young folks—but it’s also all ages that are getting involved in it.

FG1—Participant M

One participant proposed that EMA acceptability could be bolstered through explicit recruitment of MSM who identify as heterosexual or by expanding the study population to include people of all genders and sexualities who use substances:

If you were doing this survey with straight people, you might actually find out more. Like, on the down-low, how many people [are] having unprotected sex? And they may be HIV positive, and they’re using the meth, and they got a wife at home, so there’s a whole range of things that you all could cover as opposed to just asking predominantly gay and bisexual people.

FG2—Participant G

## Discussion

### Principal Findings

Few studies have explored the acceptability and feasibility of EMA among racial and ethnic minority SUMSM, and to our knowledge, this is one of the first studies to provide an in-depth, qualitative analysis that centers participants’ input and recommendations. The study participants demonstrated an overall positive reception to the EMA surveys. A high daily survey completion rate (92%) in our study mirrors the trends in recent literature supporting EMA as a substance use data collection tool among MSM [28,29,32,33]. Although race and age have previously been found to correlate with EMA engagement among MSM [32], no demographic or substance use variables were significantly associated with EMA engagement in this study with racial and ethnic minority MSM, further suggesting the utility of EMA in diverse MSM populations.

A short survey of 3 to 5 questions, delivered at midmorning each day for 7 days, and allowing a full day for response was well received by participants. Previous research has demonstrated the potential of personalized text messages to increase response rates and influence substance use and sexual behaviors [26,38,39], as bidirectional communication allows for the delivery of on-demand resources or interventions. As suggested by participants, EMA recordings may also encourage self-reflection on substance use behaviors [40]. Additional studies should evaluate the impact of EMA on substance use as well as sexual risk behaviors [26] as our findings do not corroborate the latter.

In fact, the inclusion of sexual behavior questions solely on condomless sex was not well received by some participants. As many assumed that sex under the influence of substances, often termed *chemsex*, would be condomless, survey questions asking about condomless sex appeared redundant and even stigmatizing. Although recent literature documents an epidemiologically significant rise in chemsex among MSM [41,42], successful interventions on substance use and sexual health must consider the stigma associated with substance use, HIV, and identifying as MSM as well as racism and other forms of structural violence against racial and ethnic minority MSM [43]. Our findings provide evidence that participants’ perception of judgment or stigmatization by the study design can be a barrier to participants’ engagement and honest reporting of sensitive behaviors. Future research should consider outcomes beyond condomless sex and explore whether collecting data on other sexual behaviors can help destigmatize questions regarding condomless sex. The inability to correct erroneous responses was also an important barrier to accurate data collection; future EMA studies should develop data collection systems that provide participants with the ability to make corrections to their responses to address this limitation.

Previous studies have discussed concerns over the confidentiality of substance use data, particularly if mobile devices are lost, stolen, or accessed by parties such as law enforcement [24]. Discussions in our FGs revealed that the benefits of completing the surveys on a mobile phone at any location and time, combined with abbreviation codes used for sensitive information, may afford sufficient privacy for EMA engagement. A more comprehensive understanding of the ways to maximize participants’ privacy is needed, including making data inaccessible on phones after submission.

An additional consideration of privacy and EMA engagement was reflected by suggestions to include MSM who do not identify as gay or bisexual but would otherwise benefit from these interventions. For racial and ethnic minority MSM who identify as heterosexual, sometimes labeled with the racialized term of being on the *down-low*, research studies that call for MSM participants and involve FGs or other public appearances may not be appealing due to concerns over privacy and confidentiality [44]. The use of mobile health interventions and creation of intentional safe spaces may help bridge the gap in understanding HIV transmission and other health disparities in these subgroups.

### Limitations

The limitations of this study include issues with generalizability. Owing to the small sample size, our quantitative results demonstrated relatively low power to detect small differences in compliance by demographics or substance use patterns. In addition, the EMA intervention in this study was only for 7 days; it remains unclear whether participants would have shared similar perceptions on acceptability with a longer study. Although the participants were encouraged to share experiences and opinions that differed from their peers, FG discussions tend toward normativity [45]. Furthermore, a data sample drawn mainly from prior substance use intervention research participants at the San Francisco Department of Public Health may introduce selection bias. Older individuals with substance use disorders may be more willing to volunteer for intervention research studies, which may explain why our sample that was recruited from this pool included few MSM aged under 25 years. The inclusion of only those who own a mobile phone may also have excluded lower income communities who do not have reliable access to mobile devices. Future research comparing EMA studies that do or do not provide participants with mobile devices may be warranted. Finally, this study focused solely on text message–based EMA. With the rising popularity and ubiquity of smartphones, smartphone-based apps may offer opportunities to address several of the design challenges presented in this paper.

### Conclusions

Our findings provide additional insight into the potential of EMA to collect substance use data from racially/ethnically diverse MSM. This study presents EMA as a feasible and acceptable approach that may help mitigate challenges in research conducted on stigmatized behaviors among racial and ethnic minority SUMSM. A user-centered and personalized survey design, the prioritization of privacy, and the impact of participants’ self-reflection beyond the study were important facilitators of EMA completion among our participants. Future EMA studies among racial and ethnic minority SUMSM should endeavor to retain these study elements to achieve high acceptability and compliance. Important barriers identified in our sample, such as the lack of a mechanism to correct errors and the failure to contextualize questions about sensitive topics, should be addressed to improve the acceptability of EMA approaches in this marginalized population. Ultimately, efforts to refine EMA as a study tool will help ensure equitable benefit from emerging technologies and reduce digital divides across communities disproportionately impacted by HIV and substance use.

## Appendix

Multimedia Appendix 1 Semistructured interview guide.

## Abbreviations

EMA: ecological momentary assessment
FG: focus group
MSM: men who have sex with men
SUMSM: substance-using men who have sex with men
