# Supplementary material for: Bridging the Digital Divide Among Racial and Ethnic Minority Men Who Have Sex With Men to Reduce Substance Use and HIV Risk: Mixed Methods Feasibility Study
Source: JMIR Mhealth Uhealth. 2020 Apr 29;8(4):e15282. doi: 10.2196/15282 (PMC7221651; doi:10.2196/15282)
Supplement: Multimedia Appendix 1 [file mhealth_v8i4e15282_app1.pdf]

Bridging the Digital Divide among Minority Men who have sex with men (MSM) to Reduce Substance use and HIV risk (aka EMA Study)

Overall Goal:

The purpose of this focus group is to better understand the acceptability and barriers related to a text-based set of survey questions leveraging Ecological-Momentary Assessment (EMA) data to reduce substance use and high-risk sexual behaviors among substance-using men who have sex with men (SUMSM) of color. EMA is a real-time data collection method used to collect information more immediately and more accurately from participants.

Equipment Needed:

- Audio Recorders
- Easel and paper for ground rules

Introduction Script:

Thanks for participating in the EMA Study and this focus group. We hope to learn more about the use of text message based surveys among alcohol or stimulant using MSM of color. For those of you not familiar with the acronym we use "MSM" to indicate "men who have sex with men" since not all men who have sex with men define themselves as gay. We want to understand what works well and what is challenging about using text-based surveys. Your responses, ideas, and suggestions will be very valuable to this study and for planning future programs that serve substance using MSM of color. Your insights will also help improve planning of future studies.

This session will be audio-recorded and our conversation will be confidential. The results of this focus group will not reveal responses by specific people. In other words, no names or identifying information will be attached to the responses. We will write-up all the responses, join them together, and then look for trends or patterns among all the responses. These trends, patterns, and unidentified quotes that represent how people as a whole felt will be summarized and shared with others. When we leave the focus group session you are welcome to share what you have said in the group but we ask that you respect each other's privacy by not sharing anything about what others have said. However, we cannot guarantee that everyone will keep the discussions private.

This focus group will take up to 2 hours. You may choose not to answer any question at any time.

When you enrolled in the study you were given an informed consent where you accepted that this focus group is recorded. If you do not want me to use your name, please print the name you want to be called during the session on the tent cards in front of you. I will be taking notes about the things we discuss to help myself to follow the ideas in our conversation.

Ground Rules
We want to hear from everyone in this room. In order to do so and ensure this is a safe space for your feedback, let's agree on some ground rules. Here are some we thought would be helpful:

- Anything said here, stays here
- Step Up and Step Back
  - Take turns – don't talk over people

  - Ok to pass but we want to hear from everyone
- Ok to disagree; everyone has their own opinions
- No right or wrong answers
- No judgment
- Turn off your cell phones and keep them out of sight

Are there any rules you have questions about or others you'd like to add?

Also, to make the most efficient use of all your time, we will not be taking a formal restroom break. However, feel free to step out of the room to go to the restroom at any time.

Do you have any questions about the focus group before we get started? May we begin?

[INTERVIEWER TURNS ON THE AUDIO RECORDER TO START THE FOCUS GROUP]

Let's begin with a brief icebreaker. I will ask you all go around the room, taking turns sharing the name you want to be called during the session, and also share one thing you like most about living in the Bay Area.

Focus group questions (probing questions optional):

1. **Previous experience:** Prior to this project, who all in the room has ever completed a survey using text messaging? What was that experience like for you?

2. **Intro/Overall thoughts:** Thinking back to the week that you completed the 7 days of text message-based survey questions related to substance use and sexual behaviors, what was your overall experience?
   a. What were the things you liked about it and why?
   b. What are the things you didn't like about it and why?

Next, explore barriers challenges, barriers and facilitators more broadly before getting more focused. If unexpected themes (barriers/challenges/facilitators) emerge in Q2-Q5 they can be explored more spontaneously.

3. **Explore facilitators:** Let's talk more about what you said you liked about using the text message surveys. What did you like the most? What worked well or made it easy or comfortable?

4. **Explore barriers/challenges:** What barriers might have kept you from receiving and responding to text message-based survey questions?
   a. If participants can't identify barriers, use the following examples to start a discussion:
      i. How does phone capacity (model, software update, data plan) affect your use?
      ii. Was access to wifi or having enough data or storage for texting ever an issue for you? If so, how did it affect your ability to participate?
      iii. Anything else that made the text message surveys difficult to respond to?

iv. Any ideas on how these barriers might be addressed to improve participation? Anything we can do or that participants could try?

Optional prompts if "Timing" is raised as a barrier:

Timing: *The surveys were sent around 10:30am each day and you could respond any time before midnight that day. How did the timing of the survey work for you?*

v. How did you feel about the timing we used to send surveys in this project?
vi. What time of the day would be best or make it easier to respond?
vii. What time of the day would make it more difficult to respond?
viii. How do you feel the timing influences your ability to participate?
ix. What would it be like if you were able to choose when to receive the survey?

5. **Improvements:** What solutions or improvements would you recommend for future and similar projects?
   a. What other questions should we consider asking and why?
   b. What do you think would make it work even better for you? How can we make it easier?
   c. What other technical assistance might have helped you?
   b. What forms of compensation do you recommend for people completing short surveys like those you received (in the context of a research study)?

6. **General acceptability of technology/EMA (incl barriers/facilitators):** How did you feel about using your phone for text-message based surveys?
   a. Tell me about any technical issues with your phone during the project…
      i. Texts not received, responses not sent, prompts expired, etc.? Difficulty with your involvement due to not being familiar with text-messaging, phone not working, or lost phone?
      ii. Were you able to read and understand the questions and the response options?

7. **Completion:** How did you feel about getting a survey each day for 7 days?
   a. How did you do with completing all 7 days of text based survey questions?
   b. What got in the way?
   c. What made it work?
   d. How did this affect your ability and willingness to continue?
   e. Would you respond to surveys for more than 7 days?

8. **Recall:** How accurately were you able to recall and report your substance use behaviors or sexual behaviors from the previous day?

9. **Privacy/sensitivity:** How did everyone feel about sharing information about possible substance use or sexual behaviors by text/phone?
   a. Were there challenges to keeping your responses private?
      i. Do others see or use your phone and is this a barrier to using text-messaging?
   b. What kinds of concerns if any do you have regarding confidentiality and sharing responses/data with our research team via text-message?

c. How does the subject of the question affect whether you will respond to it?
    i. For instance, when the question is asking about sensitive information, how does that affect your willingness to respond?
    ii. What kinds of questions feel very sensitive and private?
    iii. What kinds of questions make you uneasy or less likely to respond?
    iv. What, if anything can we do to help you feel more comfortable in answering the questions?

10. **Substance use effect:** What affect did your substance use on your ability to receive messages or respond to survey questions?
    a. For instance, do you feel you could forget or ignore phone; be more/less likely to disclose something private unintentionally; not be able to recall behaviors?

Thank you for your participation in our research project! Your responses will help us to better understand the needs of SUMSM of color and may lead to the development of more effective mobile device-based interventions.
